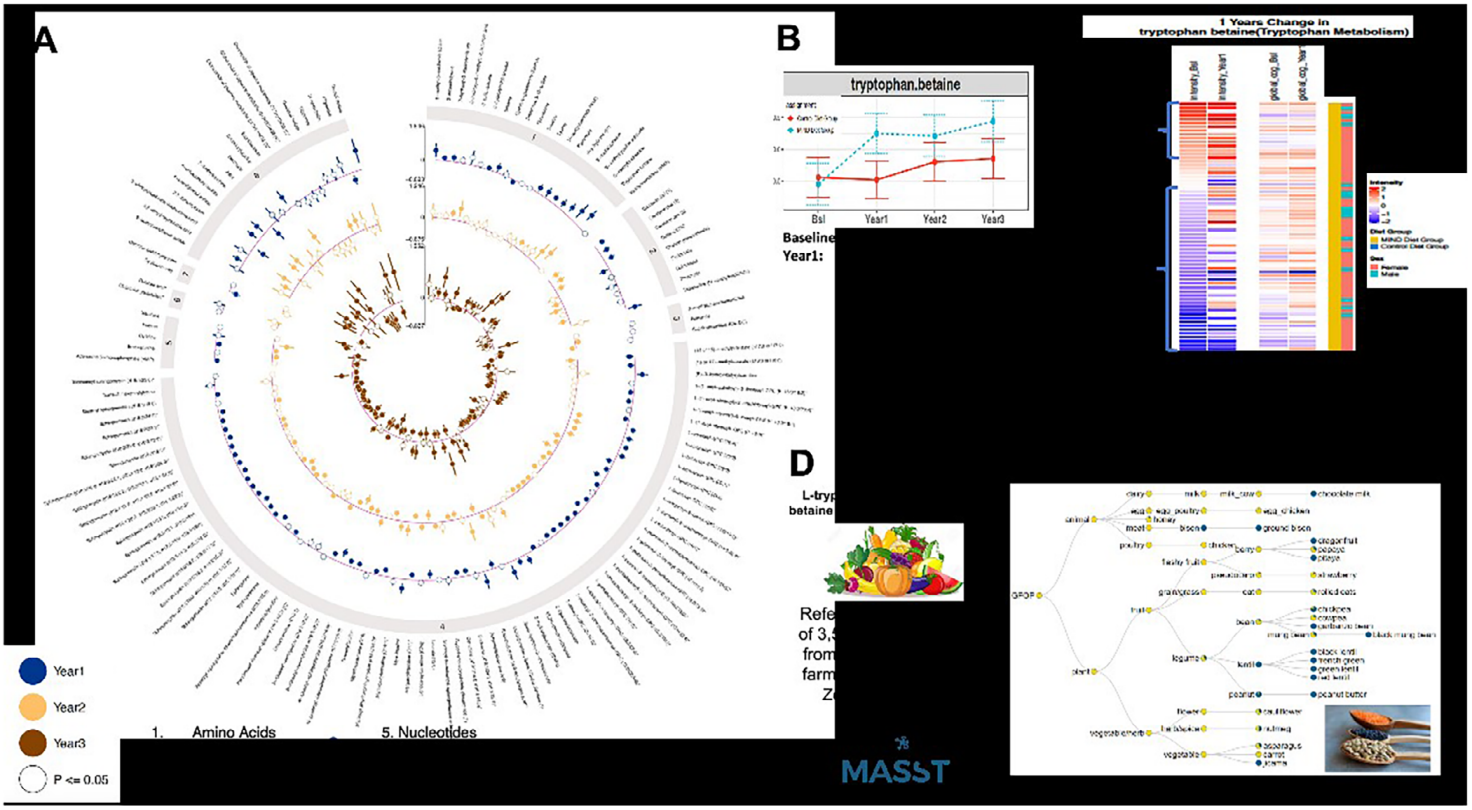# Metabolomics Analysis of the MIND Study Informs about Metabolic Benefit and Heterogeneity among Individuals‐ A Precision Medicine Approach for Diet Interventions

**DOI:** 10.1002/alz.089899

**Published:** 2025-01-09

**Authors:** Siamak MahmoudianDehkordi, Robin M Voigt‐Zuwala, Klodian Dhana, Jennifer S Labus, Ipsita Mohanty, Puja Agarwal, Martha Clare Morris, Lisa L. Barnes, Frank Sacks, Richa Batra, Gabi Kastenmuller, Ali Keshavarzian, Rima Kaddurah‐Daouk, Alzheimer's Gut Microbiome Project Consortium (AGMP)

**Affiliations:** ^1^ Duke University, Durham, NC USA; ^2^ Rush Center for Integrated Microbiome and Chronobiology Research, Rush University Medical Center, Chicago, IL USA; ^3^ Rush University Medical Center, Chicago, IL USA; ^4^ Rush Institute for Healthy Aging, Chicago, IL USA; ^5^ Goodman‐Luskin Microbiome Center, University of California ‐ Los Angeles, Los Angeles, CA USA; ^6^ UCLA Vatche and Tamar Manoukian Division of Digestive Diseases, Los Angeles, CA USA; ^7^ Skaggs School of Pharmacy and Pharmaaceutical Science, University of California‐San Diego, San Diego, CA USA; ^8^ Rush Alzheimer's Disease Center, Rush University Medical Center, Chicago, IL USA; ^9^ Harvard T. H. Chan School of Public Health, Boston, MA USA; ^10^ Weill Cornell Medicine, New York, NY USA; ^11^ Helmholtz Zentrum München, German Research Center for Environmental Health, Neuherberg Germany

## Abstract

**Background:**

Metabolomics captures net influences of exposome, diet, gut microbiome, and genome, informing about individuality and how we respond to interventions. Applications of metabolomics in pharmacology are starting to enable a Systems Pharmacology approach, where the outcome of a treatment is considered to evolve from effects on complex molecular networks, enabling insights into response variations. We bring the power of these approaches to the study of the MIND, a Mediterranean DASH diet for prevention of cognitive decline. We evaluate if metabolomics can reveal beneficial metabolic effects linked to improved cognition in all participants or subgroups of individuals.

**Methods:**

Serum samples were collected from participants enrolled in the MIND trial at the Rush University Medical Center site. Participants were randomized to either the MIND diet or control diet group for three years with study visits, cognitive testing, and sample collection occurring at baseline, Years 1, 2, and 3. A total of 746 serum samples from 243 participants were profiled using targeted and non‐targeted metabolomics, lipidomics, metagenomics, and foodomics approaches. The longitudinal effects of the diet on the metabolome were evaluated.

**Results:**

We identified metabolic signatures of participants on the MIND diet that were unique compared to the control diet. Major changes in lipid metabolism including ceramides, sphingomyelins, PUFAs, and plasmalogens were noted along with changes in energy metabolism and one carbon metabolism (Figure 1). Food components and exposome‐related metabolites were changed. For example, tryptophan betaine (lower in cognitive dysfunction) was increased in the MIND diet group with strongest effects in individuals with low levels at baseline. Additionally, glycoprotein acetyls (GlycA, an inflammation marker associated with AD, cognitive decline, reduced brain volume) was decreased in the MIND diet group compared to controls. Detailed mapping of influences on the gut microbiome are being defined and linked to changes in metabolome.

**Conclusion:**

The metabolomics data highlighted alterations in metabolism in response to MIND diet. These alterations suggest metabolic benefit for cognitive function and inflammation based on big metabolomics data in ADNI and other cohorts. The variation among individuals seen in our analysis warrants stratification of people enrolled in the MIND study before final conclusions are made on its outcome.